# Concordance study on Y-STRs typing between SeqStudio™ genetic analyzer for HID and MiSeq™ FGx forensic genomics system

**DOI:** 10.1007/s11033-023-08808-4

**Published:** 2023-10-09

**Authors:** Giulia Soldati, Stefania Turrina, Mirko Treccani, Chiara Saccardo, Francesco Ausania, Domenico De Leo

**Affiliations:** 1 Department of Diagnostics and Public Health, Section of Forensic Medicine, Forensic Genetics Lab, University of Verona, Verona, Italy; 2 GM Lab, Department of Neurosciences, Biomedicine and Movement Sciences, University of Verona, Verona, Italy

**Keywords:** MiSeq™ FGx Forensic Genomics System, SeqStudio™ Genetic Analyzer for HID, DNA typing, Y-chromosome short Tandem repeats (Y-STRs), Forensic validation and concordance studies

## Abstract

**Background:**

Massively Parallel Sequencing (MPS) allowed an increased number of information to be retrieved from short tandem repeat (STR) analysis, expanding them not only to the size, as already performed in Capillary Electrophoresis (CE), but also to the sequence. MPS requires constant development and validation of the analytical parameters to ensure that the genotyping results of STRs correspond to those obtained by CE. Given the increased frequency of usage of Y-STRs as supplementary markers to the autosomal STRs analysis, it is urgent to validate the concordance of the typing results between CE and MPS analyses.

**Methods and results:**

DNA extracted from 125 saliva samples of unrelated males was genotyped using Yfiler™ Plus PCR Amplification Kit and ForenSeq™ DNA Signature Prep Kit, which were analyzed by SeqStudio™ Genetic Analyzer for HID and MiSeq™ FGx Forensic Genomics System, respectively. For each shared Y-STR, allele designation, number of length- and sequence-based alleles per locus, stutter percentage, and the intra-locus balance of multicopy Y-STRs were screened.

**Conclusions:**

Although the number of forensic genetics laboratories that are applying the MPS technique in routine analysis is small and does not allow a global assessment of MPS limitations, this comparative study highlights the ability of MPS to produce reliable profiles despite the generation of large amounts of raw data.

**Supplementary Information:**

The online version contains supplementary material available at 10.1007/s11033-023-08808-4.

## Introduction

Capillary Electrophoresis (CE), although nowadays represents the gold standard in the forensic genetics field for the analysis of genetic length- (i.e. Short Tandem Repeats (STRs)) and sequence- (i.e. Single Nucleotide Polymorphisms (SNPs) and mitochondrial DNA) polymorphisms, used for individual identification purposes [1, 2], has undeniable application limitations mainly due to its intrinsic analytical characteristics. By using CE, the different DNA fragments of multiplex STRs are separated according to their size and identified according to the fluorophore with which they are labeled. This implies that the total number of markers detectable in a single reaction is limited by the size of the marker and directly related to the ability of the laser fluorescence detection system with which the CE system is equipped to distinguish the different fluorophores.

Current CE instrumentation allows the detection of 6 to 8 fluorescent dyes by typing a maximum of 34 STRs in a single reaction [3–7], which is not always sufficient to guarantee reproducible genetic profiling in the presence of DNA in low quantity or degraded, and mixed samples.

The analysis of SNPs or mitochondrial DNA performed in CE by Sanger sequencing allows for the typing of a single marker per reaction; consequently, to achieve an appropriate power of discrimination, it is necessary to consume large amounts of DNA, which are not always available due to the starting material from which the DNA is extracted.

Therefore, in the last decade, the interest of the international forensic genetic community has been directed to high-throughput genotyping techniques, such as Massively Parallel Sequencing (MPS) that is based on sequence fragment analysis, which can provide, in addition to length-based STR allele information, analytical data that cannot be revealed by CE analysis [8, 9].

MPS is characterized by the ability to simultaneously analyze hundreds of genetic markers, even of different types (such as STRs and SNPs) within a single reaction, and in STR loci it allows the detection of sequence variation in the flanking regions and in the repeat motif (such as isometric heterozygotes, i.e. alleles characterized by the same size but different sequences) [10–13]. In addition, MPS has enabled the development of multiplex SNPs that provide the estimation of identity, phenotypic traits, biogeographical information, and ancestry [14–16]. These relevant aspects of the MPS technique, which cannot be supplied with CE analysis, contribute to increasing the overall power of discrimination compared to the current multiplexes of STRs analyzed with CE [17], making it particularly suitable in the presence of challenging DNA samples such as complex DNA mixtures characterized by the presence of DNA discarded from multiple contributors with insufficient and/or degraded DNA [11, 18].

However, due to the complexity of the MPS procedure and its relatively high costs, this platform is present in only a few laboratories, and that limits the availability of a wide range of parameter measurements [19]. Furthermore, the commercial availability of different types of MPS platforms (e.g. MiSeq FGx Sequencing System and Ion Torrent Next-Generation Sequencing Systems), which use diverse chemical principles to detect the analyzed markers, could further contribute to slowing down the process of developing standardized criteria for the acceptability of results, precluding their routine application in forensic investigations. This limitation can only be overcome through extensive validation studies mainly aimed at comparing data between MPS and CE.

Given the current interest of the international scientific community in MPS use, these studies have mainly focused on autosomal STRs being the markers predominantly used in routine laboratory work [20–22]. However, to contribute to increasing the confidence of the results obtained with MPS, the scarce studies comparing the genotype data of gonosomal STRs (especially those on the Y chromosome), analyzed with CE and MPS, need to be implemented. Y-STRs are used when it is necessary to support the findings obtained with autosomal STRs in circumstances of male identification [23], such as in motherless paternity tests involving an alleged father and a male offspring, in the kinship analysis of half-siblings, and to reveal the male component in male-female mixtures in cases of sexual assault. MPS analysis of Y-STRs, which also highlights the sequence of the single allele and, when present, micro-variations from the reference sequence, can contribute: (1) to increase the paternity index values, (2) to explain allele inconsistencies due to single repeat mutation events between father and son and (3) in discriminating male relatives of first, second or third degree of relationship [24–26].

To purpose this aim, in this study, the concordance of the genetic profiles obtained with CE and MPS, was performed through the genotyping of the 19 Y-STR loci shared between Yfiler™ Plus PCR Amplification Kit (Applied Biosystems, MA, USA) and ForenSeq™ DNA Signature Prep Kit (Verogen, San Diego, CA) (Table S1) [27, 28], evaluating the concordance in allele designation, number of alleles per marker, percentage of stutter at each locus and the intra-locus balance for multicopy Y-STRs.

For continuity of reading, the Yfiler™ Plus PCR Amplification Kit and the ForenSeq™ DNA Signature Prep Kit are hereafter referred to as Yfiler™ Plus and FSSP, respectively.

## Materials and methods

### Sample preparation

The buccal swabs from 125 unrelated autochthonous males from North-East Italy were collected after obtaining the entitled person’s written informed consent under Italian law n.219/2017 and the approval from the University of Verona’s research ethics committee review (CARU/CARP-12). Genomic DNA was extracted from each sample using the QIAamp DNA Mini Kit (Qiagen, Hilden, Germany), and, after quantification with the Qubit dsDNA HS Assay Kit and the Qubit Fluorimeter (Qiagen), all DNA samples were normalized at a concentration of 1 ng/µl.

Additionally, the Yfiler™ Plus and FSSP kits’ DNA Control 007 and 2800 M Control DNA at a concentration of 1 ng/ µl were utilized as positive DNA controls for CE and MPS genotyping.

### Capillary electrophoresis (CE) and data analysis

The Y-STR profile of each single-source DNA sample was generated by amplification with the Yfiler™ Plus PCR Amplification Kit (ThermoFisher, MA, USA). This kit allows the amplification of 25 Y-STR markers that were genotyped with the SeqStudio™ Genetic Analyzer for HID (Applied Biosystems, Waltham, MA, USA), following the manufacturer’s instructions [27, 29] (Table S1). To maintain the signal-to‐noise ratio below the recommended level, the peak amplitude threshold (PAT) was set to 175 relative fluorescence units (RFUs), as suggested by the manufacturer of SeqStudio™ for HID.

Fragment analysis was carried out using the GeneMapper ID-X v1.6 software (Applied Biosystems) [30]. In the analysis method for the hetero- and homozygous alleles calling, the peak height threshold was arbitrarily adjusted to a minimum value of 100 and 200 RFUs, respectively. The default stutter filters, included in the YFiler Plus Analysis Files and provided by the kit’s manufacturer, were applied to set the stutter peak height values (Table S2) [27].

### Massively parallel sequencing (MPS) and data analysis

The same DNA samples were also genotyped with the ForenSeq™ DNA Signature Prep Kit using the MiSeq FGx™ Forensic Genomics system (Illumina, San Diego, CA, USA) [28, 31].

The DNA libraries were generated using the DNA Primer Mix A (DPMA) marker panel of the ForenSeq™ DNA Signature Prep kit that comprises 153 DNA markers (including 27 autosomal STRs, 24 Y-STRs (Table S1), 7 X-STRs, 94 identity-informative SNPs (iiSNPs), and Amelogenin). DNA library setup was made following the manufacturer’s guidelines. The sequencing data were analyzed by the ForenSeq™ Universal Analysis Software (UAS) (Verogen, San Diego, CA, USA) [32].

The genetic profiles with a total sample read count greater than or equal to 85,000 reads were kept, as suggested by the manufacturer. The default parameters as the Analytical Threshold (AT) and the Interpretation Threshold (IT) for all Y-STRs were set at 1.5% and 4.5%, respectively; the only exceptions were represented by the DYS389II (5.0% AT, 15% IT), DYS448 and DYS635 (3.3% AT, 10% IT) loci. To handle low coverage, UAS used a minimum of 650 reads per locus to re-calculate the AT and IT values, which were set to a minimum of 10 and 30 reads, respectively.

The default stutter filter percentages were used to correctly attribute the stutter to the sequence that differs in length from the sequence position of a parental allele and were set appropriately for each Y-STR in a range between 15% and 50% (Table S2) [32].

### Data processing

To reach the scope of this study, only the data resulting from the 19 Y-STRs common to the two amplification kits and genotyped by both CE and MPS were included in the analyses (Table S1).

The length and sequence of every allele observed at each Y-STR marker were reviewed manually using a Microsoft Office Excel sheet; then, the allelic designations provided by CE and MPS were compared.

Manual counting was used to determine the length-based alleles number per locus detected by the two techniques. In addition, alleles that exhibited sequence-based variation in MPS, either directly detected by UAS as iso-alleles at multicopy markers or indirectly by manual review, were considered additional alleles. To detect the sequence variations, those reported by the DNA Commission of the International Society for Forensic Genetics (ISFG) [33, 34] were used as Y-STRs reference sequences.

Minus and plus stutters were calculated at each locus for both CE and MPS Y-STR panels: for CE as the percentage ratio of the stutter peak height (n+/− 1 or n+/− 2nt repeat units) to the main peak height (n repeats); for the MPS as the percentage ratio of the stutter sequence intensity at (n-1) position to the parental allele intensity.

When DYF387S1 and DYS385 a/b displayed heterozygous genotypes with alleles that varied by one repeat unit, to avoid improper data interpretation, in CE the largest allele’s stutter was excluded from the calculations of the minus % stutter since it overlapped with the smallest allele’s peak, as well as the smallest allele’s stutter was excluded from the calculations of the plus % stutter since it overlapped with the largest allele’s peak.

Intra-locus balance thresholds, defined as the percentage ratio between the minor and major allele of heterozygotes evaluated in terms of RFU for CE and in the number of reads for MPS were set at 65% and 60% by GeneMapper ID-X v1.6 software and UAS, respectively [30, 32]. Therefore, when the DYS385 a/b and DYF387S1 showed heterozygous genotypes, the intra-locus balance was calculated as the ratio between the lower and the higher peak in CE analysis and as the ratio between the minimum and the maximum intensity of typed alleles in MPS analysis.

### Statistical analysis

Statistical analyses were conducted in the R environment (version 4.2.2) and plots were generated using *ggplot2* (version 3.4.1) and *ggpubr* (version 0.6.0) R packages [35–37].

Comparison between CE and MPS of stutter values at each Y-STR and intra-locus balance for DYS385 a/b and DYF387S1 loci was performed using Student’s t-test, assuming as significance threshold a *p*-value lower than 0.05 (*p* < 0.05).

## Results

To correctly interpret the data generated by MPS, the read counts per sample were always greater than 85,000, with an average overall count of approximately 93,000 reads, estimated on all 125 DNA samples.

The Depth of Coverage (DoC) was then analyzed across all the markers. Considering only the 24 Y-STRs in the FSSP panel, an average DoC of 789 reads was obtained: minimum and maximum values were observed for markers Y-GATA-H4 (213 reads) and DYS438 (3,091 reads), respectively.

Due to the lowest coverage values observed multiple times in the analysis of marker DYS392, which generated inconclusive (INC) genotype results, the comparative analysis did not consider this marker and was restricted to the 18 Y-STRs shared between the Yfiler^™^ Plus and the FSSP.

In terms of numerical values, attributed based on fragment length to the allelic variants observed at the Y-STRs shared between the two kits, their comparison revealed no significant differences in the allelic designation between CE and MPS.

**Fig. 1:**
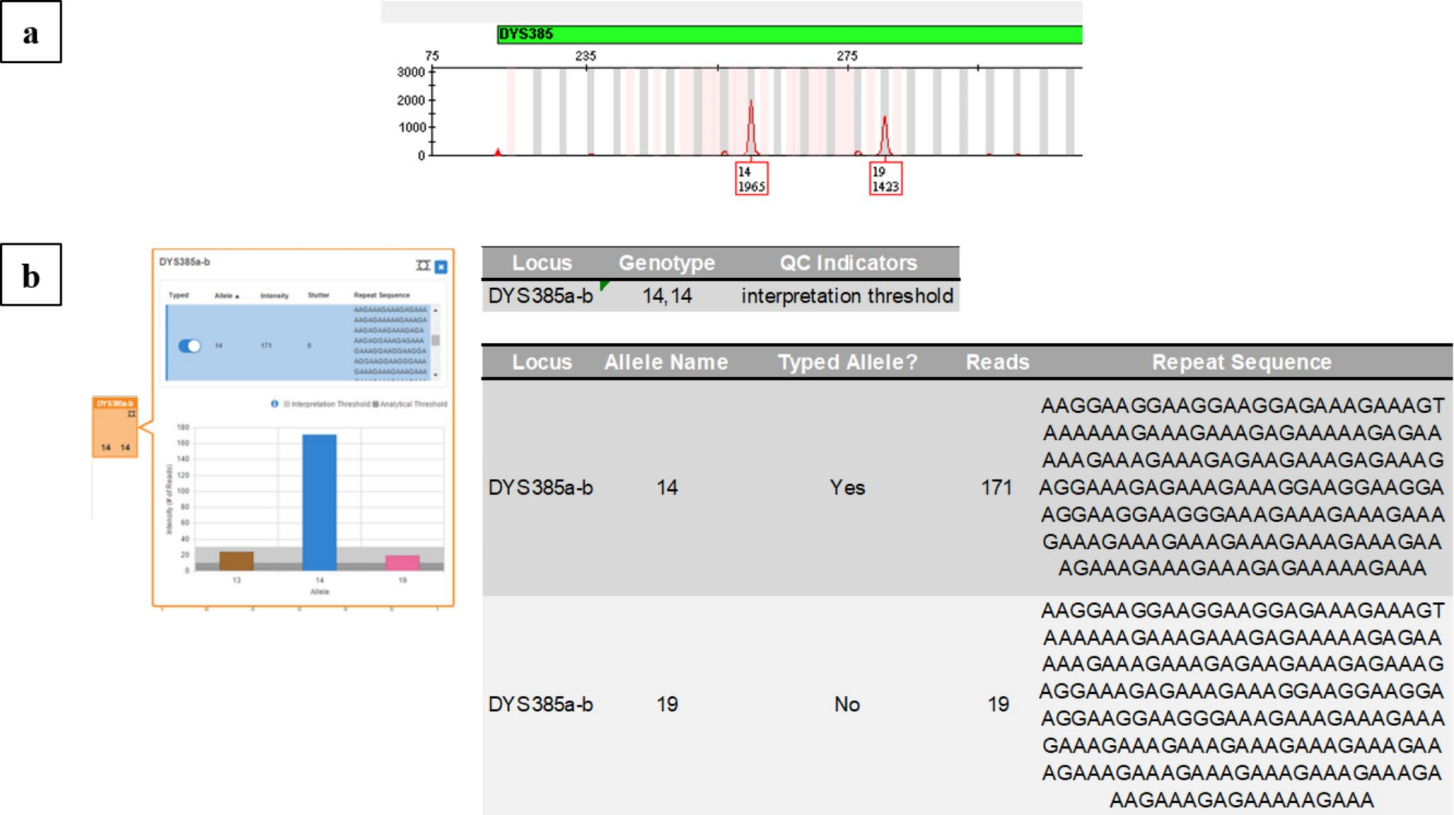
Genotypes detected at the DYS385 a/b locus by CE using Yfiler™ Plus **(a)** and by MPS using FSSP **(b)** in one sample

However, a single discordance was reported at the DYS385 a/b locus in a single sample: in this case, the CE detected a heterozygous genotype (alleles 14, 19), whereas the MPS revealed it as homozygous (alleles 14, 14). In this instance, in the *Y-STR sample report*, UAS reported a 14,14 genotype by reporting in the *QC Indicators* to pay close attention to the *interpretation threshold*; furthermore, in the bar graph, it highlighted in pink the bar related to the potential presence of an allele 19 with fewer reads than the interpretation threshold (Fig. 1). For these reasons, the observed discrepancy was considered to be only apparent and not consisting of a real difference.

Duplications at DYS448, DYS481, DYS19, and DYS576 loci, and deletions at DYS448, DYS570, and DYS576 markers, detected by CE were confirmed by MPS.

The total number of unique length-based alleles detected by CE or MPS was 119, showing full concordance between the two. Furthermore, additional sequence-based alleles, inferred from the sequence strings revealed by MPS, have been considered.

It is needed to specify that, for the multicopy Y-STR markers, such as DYF387S1 and DYS385 a/b, the iso-alleles presence (i.e. the condition of homozygosity in which the alleles have the same size but different sequences) is detectable by UAS. When homozygosity refers to alleles having the same size and sequences, but the sequence mutated compared to the reference one, and when heterozygosity refers to the two alleles with different sizes that may also exhibit mutated sequences, the sequence variants are not detectable by UAS and, therefore, it was necessary to identify them manually by the comparison with the reference sequence [33, 38].

**Fig. 2:**
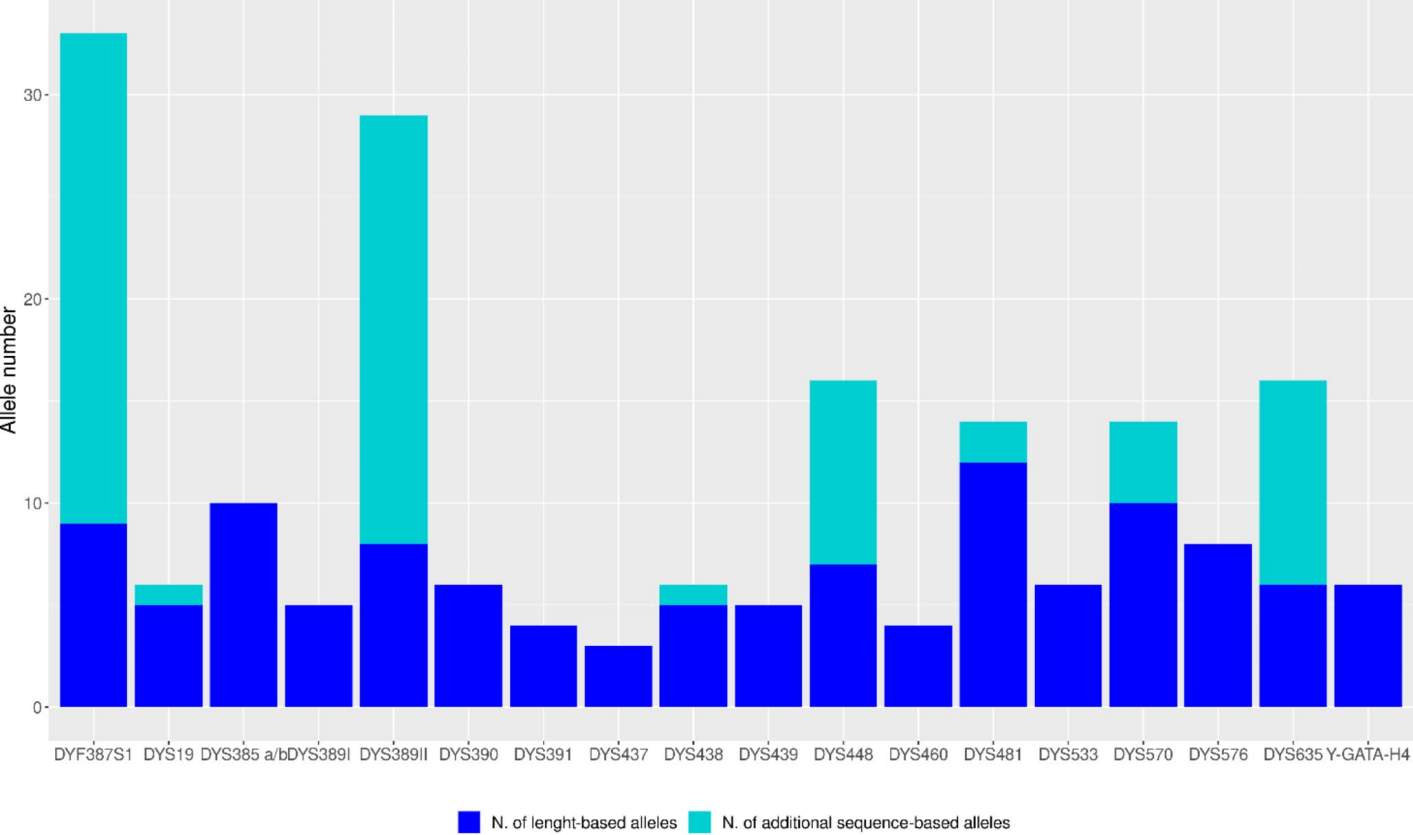
Number of length- and sequence-based alleles at each Y-STR locus shared between CE and MPS

Following this procedure, 14 iso alleles, 3 different homozygous genotypes, and 24 sequence variations in heterozygous conditions were revealed by UAS at the DYF387S1. At the DYS385 a/b marker, no different sequences were identified. In addition, 48 unique allelic sequences, different from the reference, were manually detected. These were located in 7 single-copy Y-STR loci, specifically at DYS389 II, DYS448, DYS635, DYS570, DYS481, DYS19, and DYS438. In total, 72 additional unique sequence-based alleles at 8 Y-STRs were detected. (Table S3, Fig. 2).

PCR artifacts, such as stutters, which are detectable by falling above the peak amplitude (CE) or analytical (MPS) thresholds, were following analyzed.

A total of 2,346 minus stutter (n − 1 or n − 2nt repeat units) were observed in the 125 DNA samples typed by CE using Yfiler™ Plus.

The minus stutters were revealed in 100% of cases at DYS389I and DYS635, in 99 − 90% at DYS389II, DYS390, DYS481, DYS576, DYF387S1 a, DYS438, DYS19, DYS439, DYS391, DYS437, and DYS460 loci, and in 89 − 41% at DYS570, DYS533, Y-GATA-H4, DYS385 a/b, DYS448, and DYF387S1 b markers.

**Fig. 3:**
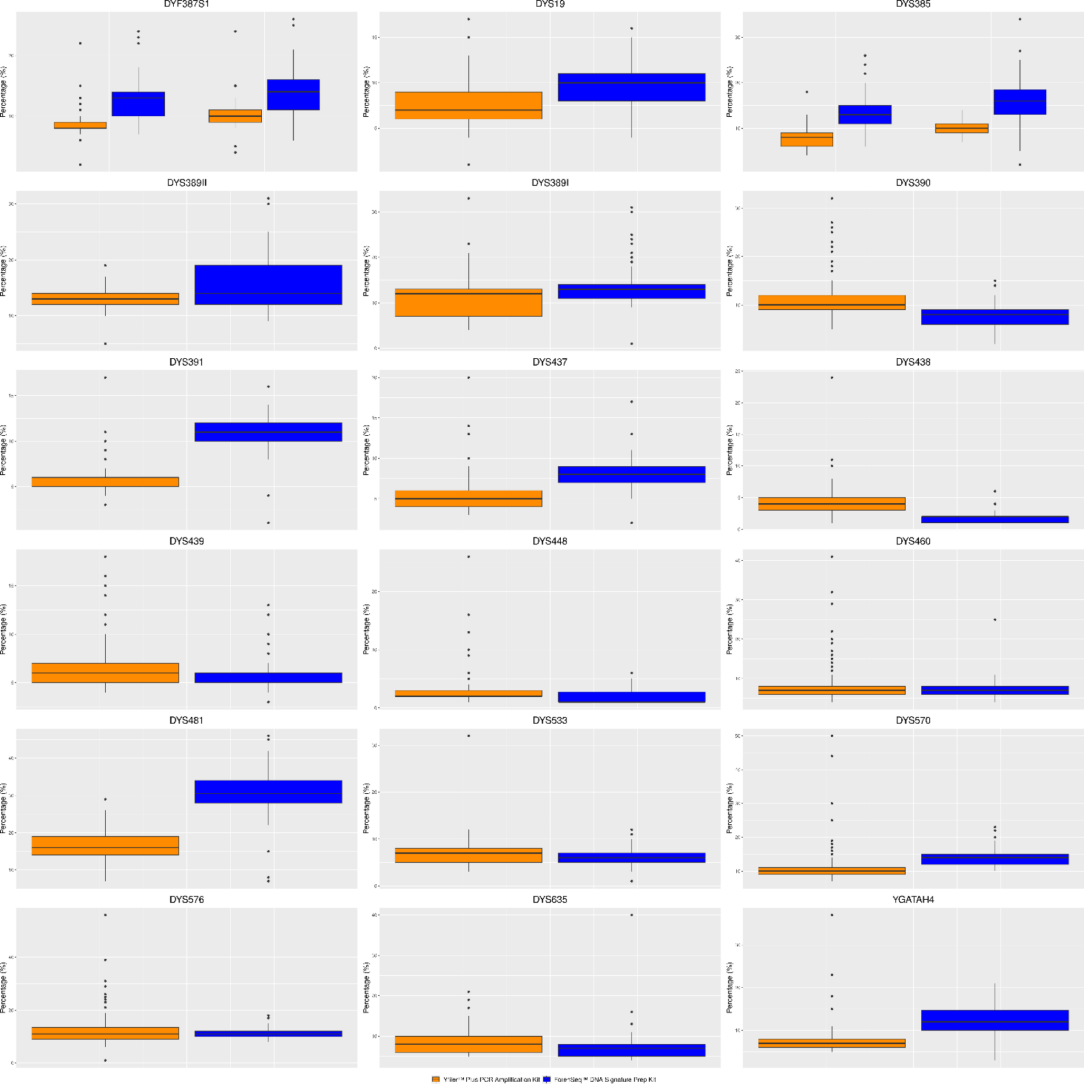
Percentage minus stutter values revealed at each Y-STR locus shared between CE (orange) and MPS (blue)

Stutter values ranged from a maximum value of 56%, estimated for the tetranucleotide marker DYS576, and a minimum stutter value of 1%, observed for DYS576, DYS19 (tetranucleotides), DYS438 (pentanucleotide), and DYS448 (hexanucleotide) loci; about 8% of the total number of observed stutters were above the set stutter threshold (Fig. 3, Table S4).

The percentage mean stutters, calculated on the stutter values detected at each Y-STR, were encompassed between maximum and minimum of 17% and 3% at DYS481 (trinucleotide) and DYS448 (hexanucleotide), respectively, resulting consistent with the default stutter filters included in the YFiler Plus Analysis Files.

A total of 29 plus stutters (n + 1 repeat unit) were observed at 6 Y-STRs (DYS389I, DYS390, DYS437, DYS439, DYS481, and DYS576).

In MPS, UAS labels each locus with a *YES* the sequence of the true allele having the maximum intensity, and with a *NO* the remaining sequences, which include the stutters (n-1 and n + 1) and the spurious sequences. Therefore, the sequence carrying the parental allele and located in position n-1/n + 1 is identified as a stutter and subsequently compared with those detected in the CE analysis.

A total of 2,010 minus stutters were observed across all the 125 DNA samples typed by MPS using FSSP.

The minus stutters were revealed in 100% of cases at DYS389I, DYS438, DYS439, DYS391, and DYS437, in 99 − 90% at DYS390, DYS481, DYS576, DYF387S1 a, DYS570, and DYS385 a, and in 87 − 30% at DYS635, DYS389II, DYS19, DYS460, DYS533, Y-GATA-H4, DYS385 b, DYS448, and DYF387S1 b markers.

The maximum stutter value of 46% was estimated for the trinucleotide marker DYS481, while the minimum stutter value of 1% was observed for DYS448 (hexanucleotide), DYS438 (pentanucleotide), DYS533, DYS389I, and DYS391 (tetranucleotides) loci (Fig. 3, Table S4).

In some cases, for the two multicopy markers DYF387S1 and DYS385 a/b, with regards to the second allele, the stutter values ranged between 21–25% and 20–34%, respectively, and exceeded the set UAS stutter filter (< 20% for both).

The percentage mean stutters, calculated on the stutter values detected at each Y-STR, were encompassed between a maximum and minimum of 31% at DYS481 (trinucleotide) and 2% at DYS438 (pentanucleotide) and DYS448 (hexanucleotide), respectively, resulting consistent with the default stutter filters included in the UAS Analysis Files.

A total of 146 plus stutters (n + 1 repeat unit) were observed at 10 Y-STRs (DYS389I, DYS390, DYS391, DYS437, DYS438, DYS439, DYS448, DYS481, DYS570, and DYS576).

The comparison between the % stutters identified at each Y-STR either with CE or MPS was performed using the Student’s t-test. For the two multi-copy loci DYF387S1 and DYS385 a/b loci the statistical analysis was performed separately on each detected allele (a and b).

No significant differences were observed at DYS460 (*p* value = 0.1723) and DYS533 loci (*p* value = 0.0573), having a *p*-value > 0.05; statistically significant differences were detected at DYS635 (*p* value = 0.0205) and DYS439 (*p* value = 0.0131) having a p-value in the range 0.01 < *p*-value ≤ 0.05; highly significant difference was revealed at DYS576 (*p* value = 0.0061) having a p-value in the range 0.001 < *p*-value ≤ 0.01; extremely significant difference were detected at all the other Y-STRs with a *p*-value < 0.001.

DYS385 a/b and DYF387S1 markers were further investigated for intra-locus balances. At DYF387S1, one sample with a tri-allelic balanced pattern (37,38,39), which was observed in both CE and MPS, was excluded from the intra-locus balance calculations.

After detecting the corresponding heterozygous genotypes at the multicopy loci DYS385 a/b and DYF387S1 by both CE and MPS, we obtained a total of 104 and 92 genotypes, respectively, and intra-locus balance values were calculated.

In CE, intra-locus balance values lower than 65% were detected in two samples at the DYS385 a/b locus and in one sample at the DYF387S1 marker; in MPS, the percentage of imbalanced read count ratio that fell below the defined intra-locus balance threshold of 60% was observed in eighteen samples at the DYS385 a/b locus and in five samples at the DYF387S1 locus.

**Fig. 4:**
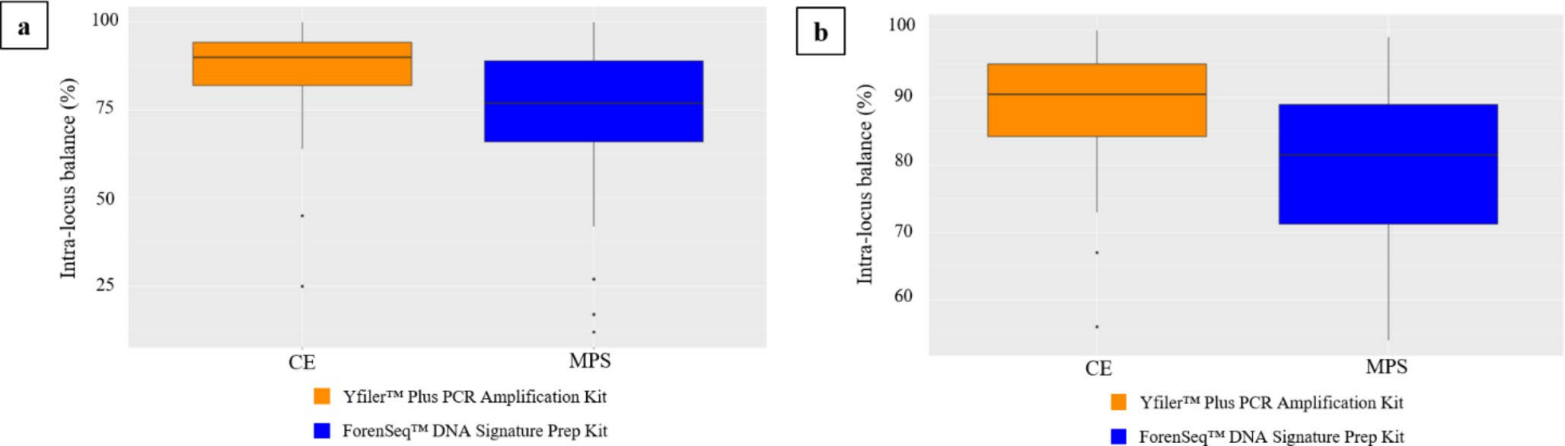
Intra-locus balance (%) at DYS385 a/b **(a)** and DYF387S1 **(b)** loci obtained from CE (orange) and MPS (blue) analysis

The Student’s t-test revealed significant differences between CE and MPS at both DYS385 a/b (CE mean = 87.26, MPS mean = 75.07, *p* = 9.711e-09; 95%CI= [8.201388;16.183227]) and DYF387S1 (CE mean = 89.42, MPS mean = 80.36, *p* = 1.894e-08, 95%CI= [6.051182;12.082151]), showing lower mean values at heterozygote genotypes revealed by MPS (Fig. 4, Table S6).

## Discussion

The MPS, introduced in the forensic genetic field more than a decade ago, due to its ability to characterize genetic markers (STRs and SNPs) by both length and sequence and to generate massive amounts of raw data, represents to date a technique that cannot be disregarded. However, as happens for all the new methodologies, the advantages and limitations tend to emerge after rigorous and accurate internal validation studies, which aim to identify the most appropriate analytical parameters to be applied for different forensic purposes. Furthermore, detailed studies are required to compare the typing results and to allow the usage of MPS in parallel with, or even in place of, the well-established CE technique [20, 21, 39].

The main goal of the current concordance study was to compare the genotyping Y-STRs results obtained from CE and MPS, considering the following parameters: allele designation, number of length- and sequence-based alleles per locus, stutter percentage, and intra-locus balance (exclusively related to multicopy Y-STRs).

Of the 19 Y-STRs shared by both forensic kits, 18 were considered, since after MPS genotyping poor or inconsistent results were consistently produced for the marker DYS392, even when sample read counts were greater than 85,000. This drawback, already highlighted by other Authors [11, 39], is also reported by the manufacturer of the FSSP kit, who advises users to be careful when interpreting marker results at DYS392 [28].

In this study, concordant allelic designation was found in all samples, with no evident discrepancy between CE and MPS in length-based allelic calling; a single exception was found in one sample, where the correct heterozygous genotype revealed at the DYS385 a/b marker by CE was instead apparently detected as homozygous by MPS.

This inconsistency was due to the MPS, which showed a low coverage (in terms of reads) at the DYS385 a/b locus, thus being unable to properly detect the allele 19 at the genotype 14,19. Despite the average read counts observed in alleles *a* and *b* of locus DYS385 being appreciable (respectively, 280 and 218), the untyped allele 19 was recognized with only 19 reads. Thus, in the bar graph, its presence was labeled in pink by UAS, since it exceeded the analytical threshold (10 reads), but not the interpretation one (30 reads), and placed this allele in a grey zone, where it is the operator who must give a correct interpretation of the obtained results. Considering that only allele 14 was commonly agreed by CE and MPS, it turned out to be necessary to screen for the presence of n-/n + stutter of the parental allele and spurious sequences, that could interfere with the correct designation of allele 19. The presence of the n-1 stutter of the allele 14 was correctly attributed in the bar graph (brown bar) by UAS, which did not report the presence of additional sequence strings characterized by n + repeat units concerning the parental allele, either in the bar graph or the *Y-STR sample report*. The analysis of the data provided only by MPS led to the assumption of a homozygous genotype 14,14, with an n-1 stutter and a hypothesized eventual n + 5 stutter (up to date, never described). Only confirmation of the MPS repeat typing of the same DNA sample and comparison with the data assumed in CE made it possible to state that there was a true allele 19 in heterozygosity with the 14, whose low coverage in reads was probably due to a binding site mutation [40] (Fig. 1).

For the 119 unique length-based alleles revealed by both techniques, a further 72 unique isometric sequence variants were detected only by MPS, either directly by UAS or indirectly by the operator (manual examination). These isometric sequence variations were discovered at eight Y-STRs, including twenty-four at the DYF387S1 locus, confirming the greater mutation rate of the marker (multicopy rapidly mutating Y-STR) [41, 42], and twenty-one at the DYS389II locus, which is a single copy Y-STR with a high average mutation rate per locus per generation.

The presence of isometric sequence variants in 60% of the unique length-based alleles of the analyzed Y-STRs can undoubtedly contribute to increasing the informativeness inferable from the analysis of these panels of markers, without necessarily increasing the number of loci to analyze [22, 43].

In CE-STRs typing results, it is common to detect the presence of peaks of several nucleotides either shorter or longer than the peak of the parental allele. These peaks, known as stutter products, are artifacts from the PCR process produced by repeat slippage during STR amplification at tetranucleotide-repeat markers, and should not exceed 15% of the parent allele [40]. Therefore, all peaks exceeding this conservative threshold value could be considered true alleles.

The presence of minus stutter at the parental allele was detected by CE and MPS in all 18 genotyped Y-STRs. The highest average value of % stutter was observed at the DYS481 locus (17% in CE and 31% in MPS), which, due to the tri-nucleotide nature of this repeat, presented an unusually high degree of stutter; however, it was always lower than the upper-limit stutter filters percentage set in the two software (Gene Mapper ID-x v1. 6 and UAS).

On the other hand, the lowest average values of % stutter were found at the DYS438 (2% in MPS) and DYS448 (3% in CE and 2% in MPS) loci, which are penta- and hexanucleotide Y-STR markers, respectively; this demonstrated that the length of the marker repeat unit has a significant effect on the stutter intensity trend, despite being influenced by the typing method used.

The presence of plus n + 1 stutter was revealed by both CE and MPS at DYS389I, DYS390, DYS437, DYS439, DYS481, and DYS576, and only for MPS at DYS391, DYS438, DYS448, and DYS570.

Considering the findings of this study, it is evident that switching from Y-STR analysis in CE to MPS leads to an increase of approximately fourfold in both the number of markers with plus stutters as well as their overall number. The stutter’s presence could make the STR mixture profile interpretation more challenging because they may mask the allelic variants in the minor contributor’s profile. Therefore, it is necessary to have a precise and accurate knowledge of the stutter percentage n- and n + at the parental allele of each STR analyzed in CE or MPS.

In the case of the Yfiler™ plus kit, stutter filter percentages n-/n + of each Y-STR were available, whereas, for the FSSP kit, the manufacturer provided unique stutter filter % values applied regardless of stutters n- or n+. Therefore, due to the lack of a specific plus stutter % filter in MPS, in this study, a real heterozygous genotype at the DYS385 a/b locus was incorrectly identified as homozygous by the MPS software, that did not recognize the imbalance of the intensity of reads at the two alleles *a* and *b*, since the reads of the allele *b* were below the IT and consistent to the % stutter filter of the locus (< 20%). Thus, the presence of the second allele with higher molecular weight was attributed by the operator.

It is known that the stutter intensity decreases when moving away from the parental allele [40], therefore, the lack of validated plus stutter metrics could direct to an incorrect genotype attribution, generating a false Y-STR profile especially when the reads of the missing true allele are below the IT.

When evaluating the intra-locus balance at DYS385 a/b and DYF387S1, based on the findings of this study, it appears that the main cause of the intra-locus imbalance detected by CE or MPS is usually due to the higher number of RFUs or reads attributed to the allele with the lower molecular weight in the heterozygous genotype of the two multi-copy loci.

Furthermore, it was found that heterozygous balance at multicopy Y-STRs is not necessarily influenced by locus coverage: for example, poor heterozygote balances observed at DYS385 a/b had higher coverage (836–1019 reads) than the average (789 reads) calculated per single Y-STR, while balanced sequencing reads in the range of 89–98% showed low coverage of reads per locus than the estimated average, as other Authors have found [21].

However, a significant difference between CE and MPS was discovered in the number of samples that showed intra-locus imbalance, demonstrating, as previously reported [26, 44–46], that CE may provide greater allelic balance and a more favorable interpretation of the data.

By searching the reference *‘Y STR MPS CE’* in the search tool Pubmed [47], 12 studies were found that compared CE with MPS on the Y-STR and were used to assess the agreement with the data deduced in the present study. A substantial consistency is evident. Specifically, poor or inconsistent results for the DYS392 locus, due to a low reads coverage, even when the total sample readings were high, were described and this was also reported by the manufacturer in the manual of the FSSP kit. [11, 28, 39, 46, 48, 49]

Concordance between CE and MPS above 99% in allele calling was confirmed on the basis of their size. The rare discrepancies that have been highlighted in other works at the DYS392, DYS393, DYS481, DYS439, and DY576 markers have been caused by the presence of SNPs in the flanking region, primers binding site mutations, and by the use of different primer sequences employed by CE and MPS systems [26, 34, 46, 48–52].

An increase of at least 40% in the total number of alleles due to sequence allele variations detected by MPS compared to those identified by CE was also confirmed by other authors [26, 46, 48, 49, 52–54], as well as the observation that the most varied allele sequences occurred in DYF387S1 and DYS389II markers [26, 34, 48, 49, 51, 53]. Concerning the presence of N-1 stutter, the information recovered from other works that had evaluated them agreed with the findings of this study: for example, the highest value of the N-1 stutter ratio was observed at DYS481, which has a trinucleotide repeat [46, 51].

## Conclusion

The MPS was introduced in forensic genetics with cautious optimism, but more than a decade later, even if its potential has been well perceived, caution has duly remained.

Unfortunately, MPS remains a technique adopted by only a few forensic genetics laboratories, and this does not allow for the background of analytical data needed to outline its application limits. In the forensic genetics field, the knowledge of benefits and limitations have the same relevance when the analytical data provided by a technique is introduced into a courtroom.

Therefore, MPS validation studies and comparison typing data studies with CE should be shared as much as possible with the international scientific community to define shared uniform guidelines relating to thresholds and forensic parameters able to guarantee a correct interpretation of the genetic profiles acquired.

A full concordance in the typing results of Y-STRs of 125 DNA samples between CE and MPS emerges from this study, although some critical interpretation issues of the data related to the high throughput of MPS and to the use of metrics, to date not completely standardized, were highlighted.

### Electronic supplementary material

Below is the link to the electronic supplementary material.

Supplementary Material 1

## Data Availability

No Data associated with the manuscript.
