# Correction: Concordance study on Y-STRs typing between SeqStudio™ genetic analyzer for HID and MiSeq™ FGx forensic genomics system

**DOI:** 10.1007/s11033-023-08908-1

**Published:** 2023-11-10

**Authors:** Giulia Soldati, Stefania Turrina, Mirko Treccani, Chiara Saccardo, Francesco Ausania, Domenico De Leo

**Affiliations:** 1 Department of Diagnostics and Public Health, Section of Forensic Medicine, Forensic Genetics Lab, University of Verona, Verona, Italy; 2 GM Lab, Department of Neurosciences, Biomedicine and Movement Sciences, University of Verona, Verona, Italy; 3 Department of Radiology, National Cancer Center, National Clinical Research Center for Cancer/Cancer Hospital & Shenzhen Hospital, Chinese Academy of Medical Sciences, Peking Union Medical College, Shenzhen, 518116 China

**Correction: Molecular Biology Reports**

10.1007/s11033-023-08808-4

In the original version of this article, the given and family name of all the authors were incorrectly swapped as “Soldati Giulia, Turrina Stefania, Treccani Mirko, Saccardo Chiara, Ausania Francesco, De Leo Domenico”.

The correct given and family name of the authors should have been “Giulia Soldati, Stefania Turrina, Mirko Treccani, Chiara Saccardo, Francesco Ausania, Domenico De Leo”.

The original article has been corrected.

